# Taking the unreal seriously: enriching cognitive science with the notion of fictionality

**DOI:** 10.3389/fpsyg.2023.1205891

**Published:** 2023-09-22

**Authors:** Pierre Gander, Kata Szita, Andreas Falck, William Hedley Thompson

**Affiliations:** ^1^Department of Applied IT, University of Gothenburg, Gothenburg, Sweden; ^2^Trinity Long Room Hub Arts and Humanities Research Institute, ADAPT Centre of Excellence for AI-Driven Digital Content Technology, Trinity College Dublin, Dublin, Ireland; ^3^Department of Special Needs Education, University of Oslo, Oslo, Norway; ^4^Department of Clinical Neuroscience, Karolinska Institutet, Stockholm, Sweden

**Keywords:** fictionality, fiction, reality-fiction distinction, pretense, cognitive science

## Abstract

Fictionality and fictional experiences are ubiquitous in people’s everyday lives in the forms of movies, novels, video games, pretense and role playing, and digital technology use. Despite this ubiquity, though, the field of cognitive science has traditionally been dominated by a focus on the real world. Based on the limited understanding from previous research on questions regarding fictional information and the cognitive processes for distinguishing reality from fiction, we argue for the need for a comprehensive and systematic account that reflects on related phenomena, such as narrative comprehension or imagination embedded into general theories of cognition. This is important as incorporating cognitive processing of fictional events into memory theory reshapes the conceptual map of human memory. In this paper, we highlight future challenges for the cognitive studies of fictionality on conceptual, neurological, and computational levels. Taking on these challenges requires an interdisciplinary approach between fields like developmental psychology, philosophy, and the study of narrative comprehension. Our aim is to build on such interdisciplinarity and provide conclusions on the ways in which new theoretical frameworks of fiction cognition can aid understanding human behaviors in a wide range of aspects of people’s daily lives, media consumption habits, and digital encounters. Our account also has the potential to inform technological innovations related to training intelligent digital systems to distinguish fact and fiction in the source material.

## 1. Introduction

People spend much of their time on activities such as movies, novels, video games, pretend play, role playing, and joint pretense in everyday conversation by using irony and humor. These phenomena all involve fictional information—information which is not intended to be about the real world and for which real-world truth conditions are irrelevant. We argue that the processing of fictional information and making reality–fiction distinctions are not odd and unusual phenomena, peripheral to human cognition. On the contrary, they are ubiquitous and as central as processing information about the real world. However, to date, cognitive science research has focused largely on cognition and behavior related to reality. Theories of perception, learning, memory, and action focus typically on how people process information about the real world. This information may turn out to be false, for example when misremembering some state of affairs or drawing an incorrect conclusion. Nevertheless, scholarly attention is generally directed toward the relation of the information to reality. Even thinking of future possibilities using one’s imagination, whose contents are in some way disconnected from facts, are directed towards scenarios that are plausible and might still be actualized in the physical world. In contrast, fictional information—which we define further below—is decoupled from reality and often does not have reference to the self. Activities featuring fictional information involve all types of cognition, such as perception, learning, and memory. Considering long-term memory as an example, theories will need to look rather different to also account for memory of fictional events not involving the self. One example is that the theory of event memory (Rubin and Umanath, 2015; Rubin, 2022) challenges classical taxonomies such as the semantic–episodic distinction (Tulving, 1972) and the explicit–implicit distinction (Squire, 1987), by including memory of events which neither involve the self nor are explicitly recalled. Another example is that traditional accounts of autobiographical memory may need to be broadened to include not only lived experience but also fictional events (Marsh and Yang, 2020; Yang et al., 2022).

The interest in the unreal does have a long—albeit narrowly confined—history in cognitive science, but without significant impact for the field as a whole. Information decoupled from reality has been studied within several areas, such as children’s pretense, narrative comprehension of fiction, and imagination. Yet, these have been mostly localized efforts, explaining particular phenomena, and researchers have not offered an integrative account beyond these specific cases. Research has yet to offer a full account of the cognitive processes underpinning fictionality and the reality–fiction distinction. Such an endeavor is also complicated by the fact that researchers may incorrectly assume that fictionality can be explained by already existing frameworks. We contend that increased and innovative research is needed on the cognition of fictionality, since, in the words of Jacobs and Willems (2018), “how exactly fiction is constructed in our brains and what distinguishes it from processing/reconstructing of facts is still an issue where research is basically fishing in the dark” (p. 147).

Contemporary uses of technology can challenge the distinction between the real and the fictional, reminding us of the importance and the urgency of understanding the processing of fictional information. Immersive technologies, such as augmented and mixed reality, are praised for their capacities for masking or manipulating the physical world, however, as such they also challenge distinguishing physical and digitally created elements perceived at a given moment. For instance, using augmented or mixed reality headsets, a user may see the surrounding world with a filter that projects a clear sky even if, in reality, the sky is cloudy. The credibility of the clear sky becomes enhanced by the fact that it is superimposed to real-world surroundings. Similarly, fully immersive virtual worlds accessed through virtual reality technology can disrupt the sensation of reality as they occupy the senses of vision, hearing, and provide haptic and embodied experiences. Presence in virtual environments together with other users—as promised by the prospective metaverse—will likely increase and replace certain parts of our everyday lives. Spending hours in virtual reality and working and socializing there has the potential to create the sense of reality in a digitally created (fictional) environment, which poses a novel challenge for cognitive science research.

**Figure 1:**
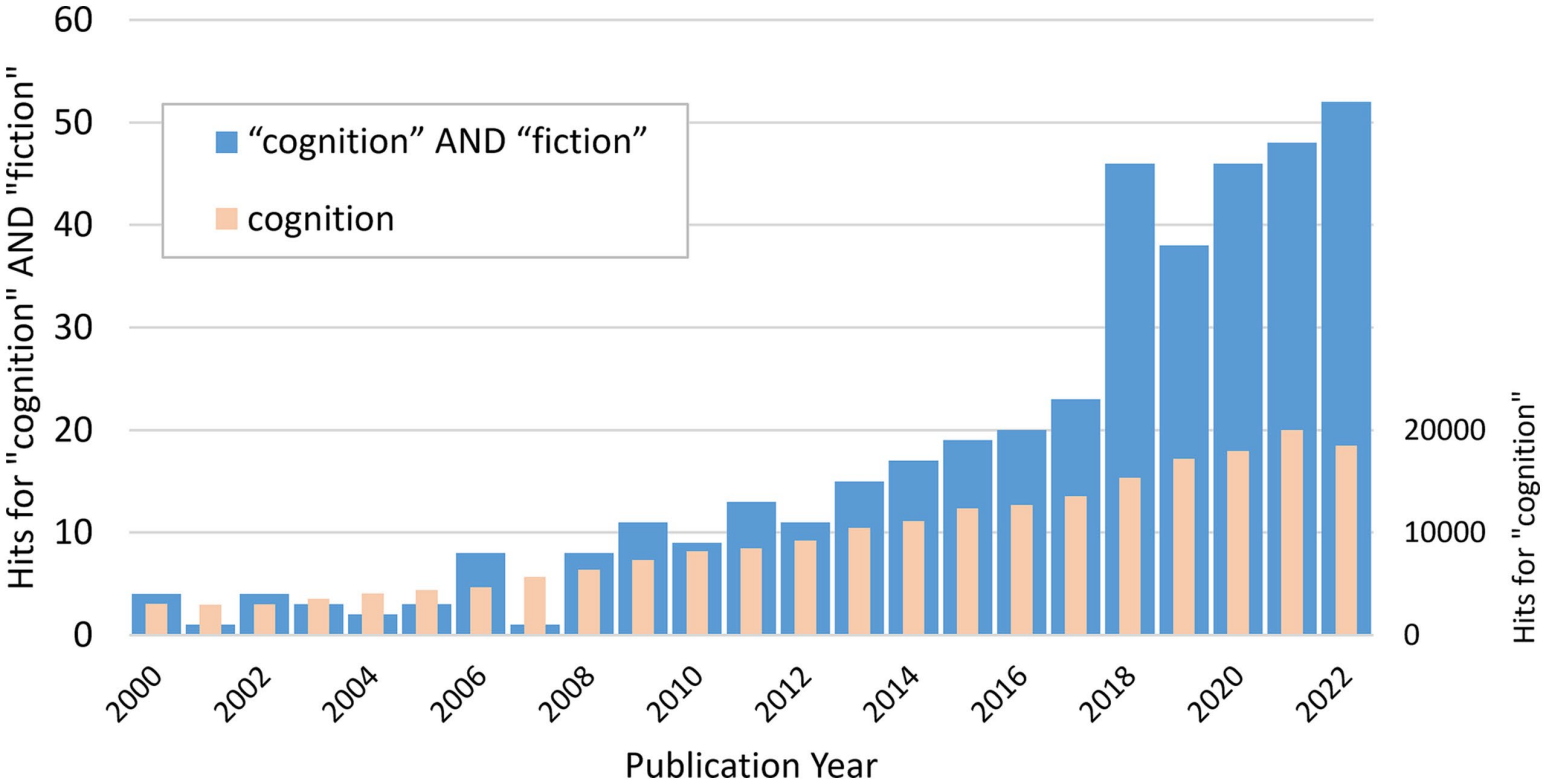
Web of science search hits for “cognition and fiction” for the period 2000–2022. Results for “cognition” (plotted on a different axis to the right) are included as comparison to a general increase of publications. The results suggest an increased interest in fiction within cognitive science during the last 5–10 years. However, we point out that this is an illustration rather than a systematic analysis of all publications in cognitive science.

So, given the ubiquity of fictionality in people’s everyday lives, it may seem strange that cognitive science has not spent more effort on theories about processing fictional information. Interestingly, we notice an increased research focus on fictionality in the recent decade. The last 5–10 years have resulted in a substantial body of publications compared to only scattered publications previously (see Figure 1). In this paper, we highlight some recent research that indicates the increased interest among cognitive scientists in fictionality. First, we will trace the roots of the focus on real-world information and knowledge in cognitive science, which we think historically has led to the neglect of fictionality. Then, we will exemplify some areas of research with a relatively long history in which fictionality traditionally has been studied. Next, we will motivate why studying fictionality in cognitive science has important theoretical and practical implications. Finally, we will close by offering a set of future challenges for the cognition of fictionality on conceptual, neurological, and computational levels.

## 2. Fictionality in cognitive science

### 2.1. The focus on knowledge and the real world

One reason why fictionality has been neglected in cognitive science, we believe, is because of its primary focus, both historically and contemporarily, on two things: (a) knowledge (of the real world) and (b) carrying out actions in the (real) world. This can be seen in definitions of the field, such as Collins (1977, p. 1) early characterization: “Cognitive science … problem areas are representation of knowledge, language understanding, image understanding, question answering, inference, learning, problem solving, and planning.” Further, the definition of Gardner (1987, p. 6) highlights the importance of answering “long standing epistemological questions—particularly those concerned with the nature of knowledge, its components, its sources, its development, and its deployment.” The focus on the real world can also be seen in contemporary characterizations of cognitive science; Mekik and Galang (2022) contend that “the core of cognitive science … [is] the study of how agents perform tasks.” However, we should point out that most definitions of cognitive science do not *exclude* fictionality, but we think that the traditional mindset of cognitive science has led to a focus on the real world, which in turn has neglected fictionality.

Notwithstanding, there are some traditions in cognitive science which consider phenomena not directly related to the real world, but as we argue, they do not really address fictionality. One example is the reality-monitoring framework, which considers how people distinguish memories of things that actually happened from things that were merely imagined (Johnson and Raye, 1981; Johnson et al., 1988). However, the fact–fiction dimension differs from the external–internal dimension since both fact and fiction can be either external or internal. Also, we note that the focus of the reality-monitoring framework is on veridicality in relation to the real world, which differs from fictionality. Other examples are false memories (Loftus and Pickrell, 1995), vicarious memories (Pillemer et al., 2015), and borrowed autobiographical memories (Brown et al., 2015). Similarly, even though these phenomena are not strictly about the real world, they are studied from a perspective of how they deviate from the real world. A final example is that planning and hypothetical thinking are not about the immediate, actual reality. However, episodic future thinking (Atance and O’Neill, 2001) and episodic counterfactual thinking (De Brigard and Parikh, 2019) still has a focus on the thinking agent’s self and the real world, considering what could happen and how things could have turned out differently. This makes these phenomena conceptually different from fictionality.

### 2.2. Going beyond knowledge of the real world

There are areas of cognitive science that focus on phenomena which do not primarily involve knowledge of the world and actions in it. Some of these areas involve fictionality or fictional information. Fictional information can be defined as some states of affairs, events, places, characters, or objects, with either internal or external origin, which is believed by a person to be decoupled from the real world. Decoupling refers to that the information is not intended to be evaluated against the real world, and real-world truth conditions and claims of existence in the real world are irrelevant (Worth, 2004; Gander, 2005; Consoli, 2018). External sources of fictional information are, for instance, novels, plays, films, pictures, comic books (Busselle and Bilandzic, 2008; Yang et al., 2022), video games, children’s pretense (Hopkins and Weisberg, 2017), role playing (Seppänen et al., 2021; Kapitány et al., 2022), oral storytelling, and pretensive elements of everyday conversation (Chafe, 1994; Brône and Oben, 2021). Fictional information could also be generated internally by imagination, such as in daydreaming. Although fictional information is commonly associated with a narrative, it is not a requirement (Consoli, 2018) and it is not limited to fiction as a storytelling genre. In biological terms, handling fictional information is mainly a uniquely human capacity, but there is some evidence that chimpanzees also perform pretend play (Kahlenberg and Wrangham, 2010; Matsuzawa, 2020). Fictional information is distinct from deception and disinformation because there is no communicative intention to deceive. Further, since our definition of fictional information requires information not to be intended to be about the real world, false information (about the world) and idealized scientific explanations (scientific models) of reality (Cartwright, 1983; Suárez, 2009), fall outside of it.

Researchers have mostly studied fiction-related situations as isolated phenomena in separate subfields with modest integration between disciplines. Explanations have been focused on aspects of the specific phenomena without extending beyond them. Fiction and imagination have been studied in philosophy (e.g., Matravers, 2014; Maier and Semeijn, 2021; Engisch and Langkau, 2022). Developmental psychologists have a long-standing interest in pretense and pretend play (e.g., Leslie, 1987; Nichols and Stich, 2000; Weisberg, 2015). Other research has studied narrative comprehension when reading fiction (e.g., Gerrig, 1993; Busselle and Bilandzic, 2008; Mar and Oatley, 2008; Oatley, 2016). This research builds on earlier traditions within the Humanities and literary studies, such as reader response theory (e.g., Fish, 1967; Iser, 1974). Out of the area of narratology, cognitive narratology has formed with a focus on how people make sense of stories (Herman, 2001). There has been an increase in experimental psychological reading research targeting the fact–fiction difference explicitly, such as studies by Altmann et al. (2014), Hartung et al. (2017), and Triantafyllopoulos et al. (2021). Further, researchers have studied the role of fiction in persuasion and misinformation (e.g., Wheeler et al., 1999; Green and Brock, 2000; Butler et al., 2012; Fazio et al., 2013; Rapp et al., 2014). Even though these examples of study areas sometimes involve integration of several disciplines (e.g., cognitive narratology integrates cognitive science and literary studies), we still find that these efforts address a specific phenomenon (stories, in the case of cognitive narratology) and that they have had little impact on general theories of cognition.

We will now highlight some recent examples of research on fictionality in cognitive science that address both the reason for fictionality and how it is cognitively processed. Dubourg and Baumard (2022a) asked in a recent article in Behavior and Brain Sciences, “Why imaginary worlds?.” Their answer is that spatial imaginary worlds, for both children and adults, have the evolutionary advantage to encourage spatial exploration. In a recent paper in Frontiers in Psychology, they consider the evolutionary advantage of another aspect of fiction, its ability to grab attention, often by containing exaggerated details (Dubourg and Baumard, 2022b). Another line of research by Kapitány et al. (2022) pointed out that pretense does not apply only to children, but also to adults. They outline the phenomenon of “pretensive shared reality” as a collaborative creation of an imaginary world, focusing specifically on table-top role playing. Other approaches from cognitive neuroscience offer yet another interesting perspective on fictionality. Abraham (2022) considers the question of how we tell apart fiction from reality. Neuroimaging studies by Altmann et al. (2014) have found differences in several brain regions when comparing fMRI activity when reading fact vs. fiction statements, which included increased activation in areas involved in complex cognitive processes such as dorsolateral prefrontal cortex and posterior cingulate cortex. Further, another fMRI study found increased amygdala activation, often associated with emotional processing, when reading more magical events in Harry Potter (Hsu et al., 2015). Finally, we highlight research on memory that has focused on the relation between memory of real and fictional events. Marsh and Yang (2020) and Yang et al. (2022) have suggested that memory of fictional events play an important role for autobiographical memory, having similar functions as lived experiences.

## 3. Why study fictionality in cognitive science?

There are several reasons why explanations of cognition should account for processing fictional, not only factual, information. Undoubtedly, the study of fiction raises fundamental issues about the nature of belief and imagination, with relevance to much of cognitive science. For example, what are the limits of people’s ability to create representations? Another example is that a general theory of human memory needs to account for not only remembering factual but also fictional information, and how these are separated in memory, since these are abilities that people use extensively in their everyday lives. Accounts of memory of fictional information have implications also for general cognition. One example of this is the relation between memories of fictional and real events and the role of memories of fictional events for autobiographical memory. In these approaches, memories of fictional events are placed in a conceptual model of general memory, providing a more complete picture of human memory, showing how memory of events may have no reference to the self (Rubin and Umanath, 2015; Rubin, 2022) and that fictional events may play important roles for autobiographical memory (Marsh and Yang, 2020; Yang et al., 2022).

The distinction between fact and fiction is also important when applying theory to real-world situations. Generally, factual information should update people’s knowledge of the real world and guide goal-directed behavior, while fictional information should not. In some cases, fact and fiction are confused, so that a person takes fiction as fact, either at encoding during comprehension, or at later retrieval of information. In this way, fiction can be a potent source of misinformation, especially in the form of narratives. For example, concerns have been expressed about the danger that jurors’ perceptions of the US justice system are shaped by fictional television shows such as *Law and Order* (Shniderman, 2014). Theories that specifically address how the reality–fiction distinction is cognitively processed are needed to explain these cases of confusion. But this blurred line between fact and fiction also allows for learning facts from fictional sources, such as with children’s pedagogical books or historical novels (Marsh et al., 2003; Hopkins and Weisberg, 2017). Understanding if, when, and how such transfer takes place becomes relevant for any account of human cognition, which also has implications for real-world scenarios in areas such as education and media policymaking.

Finally, the cognitive processing of fictionality is also important for technological applications. One example is that future social household robots may be required to handle fictional information, because they will be exposed to entertainment media in home environments. Considering that such robots have a role to alert in case of danger, fictional information may pose a problem. For instance, a robot perceiving an action film showing on a screen or perceiving children playing with toy guns could result in that the situation is incorrectly considered dangerous or threatening. The robot would need the ability to process fictional information as distinct from factual information in order to act appropriately in the situation. To our knowledge, no robotic system currently takes fictional information into account in either perception or memory. Another example is that virtual agents and conversational AI systems access the vast information available on the Internet, but are not always able to qualify information as factual or fictional. For example, large language models such as ChatGPT summarize information from various sources, and sometimes “hallucinate” factually inaccurate answers to factual questions (Maynez et al., 2020; Susarla et al., 2023; Tam et al., 2023). In training these models, AI researchers need to consider how fiction is presented, and that fictional information is often presented as factual without deceptive intent. Beyond engineering aspects, these applications also include design issues relevant to fields such as human–computer interaction, interaction design, and human–robot interaction. Within design research, there is another way in which fictionality is taken seriously, namely in *design fiction*. Here, fictional scenarios and personas are used during the design process to explore future possibilities and to help understand requirements, values, and implications of technology (Bleecker, 2009; Lindley and Coulton, 2015). Well-known examples include the movie *Blade Runner* and George Orwell’s novel *1984*. By creating a story world, insights into non-existing technologies can be gained which would not be possible otherwise.

To conclude, there are several reasons to include the notion of fictionality in cognitive science. Without considering fictional information, theories of cognition are lacking, such as memory theory. In everyday life, mistaking fiction for fact can have highly adverse consequences, but learning of facts from fictional sources can also be beneficial. In AI and robotics, the neglect of the fact–fiction distinction could lead to inappropriate behavior and presentation of inaccurate information to human users. Finally, insights into future technological applications of cognitive science can be gained using design fiction.

## 4. Three future challenges

Although research on cognition and fiction is increasing, we see that the study of the cognitive foundation of fictionality presents some important future challenges for research. We outline three challenges below: (a) conceptualization, (b) the reality-fiction distinction, and (c) modeling in artificial systems. We regard the three challenges as distinct but also feeding into each other so that research on one challenge can benefit the others.

One fundamental challenge is how to conceptualize fictionality in relation to cognition. What is the scope of application? Should it be applied only to fiction-related experiences (e.g., movie watching) or also actions (e.g., pretend play)? In the case of memory of fictional information, fictionality needs to be explicated in relation to other memory characteristics, such as belief, confidence, plausibility, realism, vividness, and psychological distance. Further, researchers should consider whether fictionality is best seen as a dichotomy of fact and fiction (Yang et al., 2022) or as a continuum (Woolley, 1997)—perhaps analog to graded belief (Dietrich, 2022). It is also an open question if it is useful to consider various kinds of unreal under the same concept, such as fiction, magic, religion (Boyer, 1997), and myth (Eliade, 1968). In these considerations, it becomes clear that also cultural aspects of fiction and related phenomena need to be taken into account. For example, the nature of the distinction between the real and the unreal may vary across cultures. In some cultures, the distinction may be more important or clear, while in others less so. Finally, the issue of the conceptualization of fictionality relates to the question of how to best characterize experiences and cognitive processing with virtual and mixed-reality technology. Should these be treated as real or fictional, or perhaps as a third kind?

Another challenge is to account in detail for the underpinnings of how the cognitive system handles the reality–fiction distinction. Exactly how is this distinction made in online comprehension as well as during memory retrieval? Answers to these questions are needed in order to account for both how people can keep reality and fiction apart in order to carry out appropriate goal-directed behavior in the real world, but also how misinformation arises from reality–fiction confusion when fiction is taken as fact. Current approaches do not sufficiently explain the reality–fiction distinction. The source-monitoring framework can only partly account for the fact–fiction distinction, although it has previously been considered a sufficient explanation (e.g., Potts and Peterson, 1985; Potts et al., 1989; Gerrig and Prentice, 1991; Johnson et al., 1993; Marsh et al., 2003). The framework needs to be supplemented for at least three reasons: (a) a single source can contain both fictional and factual information (e.g., a children’s educational book with fantasy elements), (b) some fictional information would not be associated with source information (e.g., knowledge about unicorns), and (c) mere identification of a source does not account for how the cognitive system handles fictional information differently from factual information, and the consequences this has for cognition. Further, neither the reality-monitoring framework (Johnson and Raye, 1981; Johnson et al., 1988) nor processing fluency (Fazio et al., 2013; Rapp et al., 2014) are good candidates for an explanation of this ability, since they cover only a limited number of cases—they rather address dimensions that are orthogonal to the reality–fiction dimension. Instead, a more elaborate account is needed in order to explain the reality-fiction distinction. The topic needs be addressed across multiple levels of organization, incorporating (at least) neural, cognitive, and cultural aspects. Insights into the reality-fiction distinction can also be gained by studying it from developmental and pathological perspectives.

A final challenge is how future applications of detailed accounts of cognition and fictionality could be modeled in artificial systems. Such implementations could be for the purpose of modeling human cognition (directly related to the second challenge above), or for more need-based engineering purposes. Systems designed to solve tasks may be non-interactive (such as processing of big data, for example, the training of generative AI models), or interactive (such as chatbots or social robots operating in human environments in which they need to handle fictional information).

## 5. Conclusion

We have argued that fictionality, although ubiquitous in people’s lives, is a neglected theme in cognitive science. We pointed out that cognitive explanations of fictionality are pressing and highlighted a number of recent approaches that tackle questions on processing of fictional information and the reality–fiction distinction. Such approaches not only relate to specific phenomena, such as narrative comprehension or imagination, but have impact for general theories of cognition for both humans and in intelligent artifacts. There are implications also for applied cognition in real-world settings as well as engineering technological applications. We highlighted future challenges for the cognitive study of fictionality on conceptual, neurological, and computational levels. Taking on these challenges requires interdisciplinary collaboration across all branches of cognitive science. Areas such as developmental psychology, narrative comprehension studies, memory research, and philosophy need to share research findings and integrate accounts, in order to answer the questions outlined here.

## Data availability statement

The original contributions presented in the study are included in the article/supplementary material, further inquiries can be directed to the corresponding author.

## Author contributions

PG wrote the first draft of the manuscript. PG, KS, AF, and WT revised the manuscript. All authors contributed to the article and approved the submitted version.
